# Examining Language Switching and Cognitive Control Through the Adaptive Control Hypothesis

**DOI:** 10.3389/fpsyg.2020.01171

**Published:** 2020-07-24

**Authors:** Gabrielle Lai, Beth A. O’Brien

**Affiliations:** ^1^Centre for Applied Behavioural & Social Sciences, Temasek Polytechnic, Singapore, Singapore; ^2^National Institute of Education, Nanyang Technological University, Singapore, Singapore

**Keywords:** language switching, Adaptive Control Hypothesis, cognitive control, word-switching, inter-sentential switching, intra-sentential switching, interactional contexts

## Abstract

Increasing evidence suggests that language switching is a distinct form of bilingual language control that engages cognitive control. The most relevant and widely discussed framework is the Adaptive Control Hypothesis. This theoretical framework identifies language switching to be a key aspect of bilingual language control. It proposes that bilinguals’ engagement in three different types of interactional contexts (single-language context, dual-language context, and dense code-switching context) confers adaptive effects on cognitive control processes. These contexts differ in the presence of both languages and how language control is exercised. The model makes predictions about behavioral outcomes associated with these contexts. This study is a novel attempt to test for the model’s assumptions, predictions, and its interactional contexts. It seeks to examine the relationship between language switching behaviors, reported bilingual interactional contexts, and verbal and non-verbal cognitive control through this theoretical framework. Seventy-four English–Mandarin young adult bilinguals were measured on their self-reported engagements in the different interactional contexts and production of word and sentential language switches through experimental language switching tasks (alternating, semi-cued, and uncued switching). Cognitive control processes in verbal and non-verbal goal maintenance, interference control, selective response inhibition, and task engagement and disengagement were measured. Overall, partial support for the model was observed. Higher reported engagement in the dual-language context was positively but not uniquely related to cognitive engagement and disengagement on verbal tasks. Non-verbal goal maintenance and interference control, on the other hand, were related to uncued inter-sentential language switching. However, the distinction of the model’s three interactional contexts might not be evident in a multilingual society, as findings suggest that there is fluidity in bilinguals’ interactional contexts. Current findings reveal the complex interaction of language switching with distinct domains and cognitive control processes. This study is significant in testing an influential bilingual language control model.

## Introduction

Language switching is a distinctive capability that reflects cross-linguistic activation and a systematic control of two languages (Kroll et al., 2015). Neural studies have shown considerable shared overlap of neurocognitive mechanisms between bilinguals’ language switching and cognitive control processes (e.g., Abutalebi and Green, 2008, 2016, Weissberger et al., 2015). Behaviorally, differences in language-switching practices have been found to be associated with cognitive control processes such as monitoring and switching (e.g., Costa et al., 2009; Soveri et al., 2011; Hartanto and Yang, 2016; Verreyt et al., 2016; Henrard and van Daele, 2017; Barbu et al., 2018). These findings lend increasing evidence in demonstrating language switching to be a distinct form of bilingual language control that necessitates and engages non-linguistic cognitive control operations.

The relationship between language switching and cognitive control has been discussed more thoroughly in a theoretical framework, known as the Adaptive Control Hypothesis (ACH, Green and Abutalebi, 2013). Central to this framework, language switching is argued to be a significant aspect of bilingual language control that implicates non-verbal cognitive control processes in its engagements. It proposes that neural and cognitive control adaptations are involved through the types of interactional contexts (recurrent patterns of conversational exchange) that bilinguals primarily engage in on a day-to-day basis.

The Adaptive Control Hypothesis considers three types of interactional contexts: Single-language contexts, dual-language contexts, and dense code-switching contexts. These three interactional contexts differ in the degree of language control that is required during language switching based on two key aspects. The first aspect is in the presence (or absence) of both languages. This pertains to the degree of exposure and use of both languages in the bilinguals’ external linguistic environment. The second aspect is how interference is resolved. This is related to how bilinguals exercise internal linguistic control and switch between their languages. The model discusses this to be reflected in the types of code-switches depending on the level of linguistic integration. It assumes that inter-sentential switches involve greater levels of inhibitory control than intra-sentential switches.

In the single-language context, one language is used in one environment and the other language is used in another distinct environment (e.g., L1 at home and L2 in school). In this context, both languages are mostly kept apart in bilinguals’ interactions, and language switching is infrequent (i.e., low presence of both languages). In a dual-language context, both languages frequently co-occur and language switching is frequent (e.g., L1 and L2 are used at school). Different languages are typically used with different speakers and language switching may occur within a given conversation, but not within the same utterance (inter-sentential switching). As the production of both languages is kept apart, language control is argued to be high due to the state in which both languages are controlled (competitive mode) (Muysken, 2000; Green and Wei, 2014; Green, 2018). In a dense code switching context, both languages are also present, and speakers tend to mix their languages in the course of a single utterance and adapt words from one of their languages to fit in with the other (intra-sentential switching). In this form of language switching, language control is argued to be low as both languages are used opportunistically and are in a cooperative (rather than competitive) state. Based on the differences in linguistic control demand that each interactional context necessitates, the hypothesis proposes that adaptive and distinct effects on cognitive control processes will be observed within bilingual speakers who engage in these respective interactional contexts. The Adaptive Control Hypothesis makes further predictions about the linguistic and cognitive control outcomes associated with bilinguals’ primary engagement in these different interactional contexts (see Table 1 for predictions). The focus of this study is an exploration of these predictions.

**TABLE 1:** Control components proposed by the ACH with effects of bilingual interactional contexts and cognitive control task measures.

**Control processes proposed in the ACH**	**Interactional contexts**			**Cognitive control tasks**
	**Single-language context**	**Dual-language context**	**Dense code-switching context**	**Measures for the verbal (V) and non-verbal (NV) tasks**
Goal maintenance	+	+	=	V: Stroop mixing cost: Difference between incongruent trials (mixed block) and incongruent trials (pure block) NV: Global–Local mixing cost: Difference between local incongruent trials (mixed block) and local incongruent trials (pure block)
Interference control: conflict monitoring	+	+	=	V: Stroop: RTs for incongruent trials (mixed block) NV: Global/Local: RTs for local incongruent trials (mixed block)
Interference control: interference suppression	+	+	=	V: Stroop effect: RT difference between incongruent trials (pure block) and congruent trials (pure block) NV: Global/Local conflict effect: RT difference between incongruent local trials (pure block) and congruent local trials (pure block)
Selective response inhibition	=	+	=	V: Stroop: Overall RTs on incongruent trials (pure block) NV: Global/Local: Overall RTs on local incongruent (pure block)
Task engagement and disengagement	=	+	=	V: Stroop switch cost: RT difference between switch and repeat trials (mixed block) NV: Global/Local switch cost: RT difference between switch and repeat trials (mixed block)

*+ indicates that the context increases the demand on that control process; − indicates that the context is neutral in its effects (salient cue detection and opportunistic planning were also included in the ACH control processes, but these were not tested here) (Green and Abutalebi, 2013). V, verbal task; NV, non-verbal task and measures used in current study per control process.*

The predictions within the ACH model pay specific attention to the dual-language context, due to the highest linguistic and cognitive control that is demanded within it. In the dual-language context, the process of *goal maintenance* is activated when the bilingual must establish and maintain a task such as speaking in one language rather than another (Green and Abutalebi, 2013). This maintenance requires interference control processes (*interference control*), which is proposed to be related to two control processes of *conflict monitoring* and *interference suppression*. The process of detection of salient cues is also important in successful communication as the detection of changes in the interactional context (e.g., arrival of another speaker) might require the bilingual to switch and use their other language (*salient cue detection*). The bilingual has to prevent themselves from continuing to speak in the current language, using selective response inhibition (*selective response inhibition*). This then triggers the need for the bilingual to disengage from the current language. In order to switch languages effectively, the bilingual will have to disengage from the previous language and activate the new one (*task engagement and disengagement*). Accordingly, the dual-language context is proposed to be associated with cognitive monitoring and inhibitory control processes.

By contrast, in the single-language context, the ACH model predicts that effects will be mainly observed in cognitive monitoring processes of *goal maintenance* and *interference control* (Green and Abutalebi, 2013) (Table 1). In this context, bilinguals’ languages are kept apart, and there is lesser demand on linguistic control. In the dense code-switching context, distinct effects on opportunistic planning control processes are proposed (opportunistic planning). By using whichever language is most readily available, bilinguals adapt words from one language to fit into another and languages are used opportunistically (intra-sentential switching). However, this does not mean that speech in the dense code-switching context is not cognitively demanding.

In an innovative attempt to examine the Adaptive Control Hypothesis, Hartanto and Yang (2016) compared young-adult bilinguals who differed in their engagement in the single-language context and dual-language context, on a non-verbal task-switching paradigm through the color-shape task. Bilinguals were classified into these contexts based on the extent to which they reported using two languages within the same context. Findings from their study indicated that dual-language context bilinguals demonstrated smaller switch costs and were significantly faster in switch trials as compared to those in the single-language context bilinguals. Further, they found that bilinguals in the dual-language context demonstrated faster reaction times (RTs) (efficiency) on switch trials. Notably, this study also revealed that higher reported inter-sentential switching was correlated with smaller switch costs (efficiency). On the other hand, intra-sentential switching positively predicted switch costs (in the opposite direction), demonstrating that a greater reliance on language switching within sentences was likely to diminish executive control efficiency. The results from this study were interpreted to suggest initial evidence for the Adaptive Control Hypothesis, in showing that bilinguals’ engagement in dual-language environments where both languages are frequently used (i.e., dual-language context) could influence cognitive control efficiency. Their results also suggested seminal evidence associating different types of language switches with cognitive costs and efficiency.

Support for particular effects of the dual-language context is also suggested in a later study (Henrard and van Daele, 2017). Professional interpreters and translators, who differed in the language control demands (i.e., time pressure) that they face in a dual-language environment, were compared with monolinguals on various aspects of cognitive control. Results showed that as compared to monolinguals, both interpreters and translators demonstrated efficiency in cognitive flexibility and inhibition. More notably, this study showed an “interpreter advantage.” Interpreters outperformed monolinguals on all cognitive measures and were more efficient than translators in processing speed and inhibition. These results were suggested to lend evidence for the dual-language context, and further demonstrate the co-varying effects of bilinguals’ engagement in linguistically demanding environments on non-verbal cognitive control efficiency. These studies add to the growing body of evidence associating cognitive control efficiency with bilinguals’ reported engagement in dual-language environments, in which both languages are present and used frequently. In relation to the Adaptive Control Hypothesis, these lines of evidence support one aspect of the model, which suggest that the presence (or absence) of both languages and bilinguals’ frequent exposure to them could influence cognitive control.

In examining the other aspect of the Adaptive Control Hypothesis, which pertains to how language interference is resolved (i.e., types of sentential switches), discrepant findings are observed. In Hartanto and Yang (2016), higher reported intra-sentential switching predicted cognitive costs (poorer efficiency), while inter-sentential switching predicted cognitive efficiency. However, these findings were directly contrasted in another study, which measured bilinguals’ sentential switching in an ecologically more valid manner through a frequency judgment task (Hofweber et al., 2016). In this study, two groups of German–English bilingual adults, who differed in their dense code-switching behaviors, completed the frequency judgment task. They were asked to imagine having a conversation with another bilingual friend, and to rate the frequency with which they would most likely encounter a series of code-switching utterances. This study found that the bilingual group who reported engagement in more dense code-switching (intra-sentential switches) demonstrated non-verbal inhibitory control advantages on the Flanker task, particularly in high conflict monitoring conditions. Correlation analyses also revealed that a higher frequency of dense code-switching was positively associated with non-verbal conflict-monitoring abilities. In contrast to Hartanto and Yang (2016), this study did not find any association between alternation (similar to inter-sentential switching), with non-verbal cognitive control efficiency. These findings led the authors to argue that dense code-switching, in which bilinguals switch between their languages within utterances (intra-sentential switching), is a natural type of language production among bilingual populations. They argue that while dense code-switching may engage global forms of inhibition to a lesser extent, dense code-switching may challenge and train cognitive monitoring processes. These findings could highlight the methodological sensitivities in measuring sentential language switching behaviors, particularly in relation to cognitive control. Significantly, the finding with cognitive monitoring efficiency challenges the Adaptive Control Hypothesis on its assumption that dense code-switching behaviors do not have effects on cognitive control processes.

This is further demonstrated in more recent neural and behavioral studies that have experimentally induced the dense code-switching context (e.g., Blanco-Elorrieta and Pylkkänen, 2017, 2018; de Bruin et al., 2018). In these studies, when bilinguals are given the freedom to switch between their languages and use them voluntarily, it is observed that language switching is minimally demanding, and perhaps even beneficial, due to the intuitive way that bilinguals naturally use both their languages. For instance, in Blanco-Elorrieta and Pylkkänen (2017), adult bilinguals engaged in a phone conversation with bilingual and monolingual interlocutors and had to name pictures in a language suitable for communicating with the interlocutors. Results showed that the neural signatures of effortful language switching—increased anterior cingulate and prefrontal cortex activation—disappeared when bilinguals engaged in voluntary language switching (dense code-switching context). In another study, no additional cognitive costs were imposed for word items when bilinguals were allowed to switch between their languages and name pictures in whichever language was easier for them (Kleinman and Gollan, 2016). These studies, which demonstrate neural and behavioral efficiency, at least at the word level, show how uncued and naturalistic language switching is linguistically effortless. Thus, the premise of naturalistic language switching behavior involving cognitive control comes into question.

In view of these mixed findings, the cognitive effects associated with how bilinguals manage and exercise internal linguistic interference, particularly with voluntary sentential-level language switches, remain unclear. This is due to the dearth of studies that have examined the cognitive effects associated with sentential-level switching. Current studies have mostly relied on self-reports or subjective ratings as measures of bilinguals’ sentential-level language switching behaviors (e.g., Hartanto and Yang, 2016; Hofweber et al., 2016), and few studies have attempted to experimentally induce bilinguals’ naturalistic verbal production sentential language switching utterances (see Kang and Lust, 2019; Hofweber et al., 2020). The current study is novel in its attempt to induce bilinguals’ naturalistic verbal production of word and sentential language switching behaviors through experimentally varying language switching demands (cued switching, semi-cued switching, and uncued switching). It aims to examine the association between the different types of language switches in these various contexts with a range of cognitive control processes that are proposed within the Adaptive Control Hypothesis (Green and Abutalebi, 2013).

Based on current evidence, it is observed that support for (or lack of) the model’s interactional contexts have mostly been inferred, and its predictions have not been directly examined (e.g., Hartanto and Yang, 2016; Hofweber et al., 2016; Blanco-Elorrieta and Pylkkänen, 2017; Henrard and van Daele, 2017). It is also observed that there has not been a study that has tested for the three interactional contexts, and the linguistic and cognitive control predictions made with regard to bilinguals’ engagement in them (Table 1). Although the hypothesis assumes a theoretical classification of the three interactional contexts (single-language context, dual-language context, and dense code-switching context), it also discusses the likelihood that there is fluidity in bilinguals’ natural communicative environments and language ecologies. It is likely that bilinguals may engage in these different interactional contexts to varying degrees and may not find themselves in a specific interactional context. This echoes similar views, which argue that bilingualism is a continuous variable and should not be viewed dichotomously or categorically (e.g., Luk and Bialystok, 2013). Nonetheless, the model proposes that bilinguals’ primary engagement in these different types of interactional contexts could have distinctive effects of cognitive control due to the linguistic and cognitive demands that each interactional context might implicate. Accordingly, this study is unique in its attempt to examine the ecological validity of these interactional contexts within bilinguals. It also seeks to examine how bilinguals’ individual variations of engagement in these interactional contexts are associated with various cognitive control processes.

For this study, the verbal Stroop task and non-verbal Global–Local tasks were used to examine cognitive control. Both tasks were selected as cognitive task measures due to their similarity of cognitive processing demand (i.e., stimulus–stimulus inhibitory control). For both tasks, potential conflict occurs between the two levels that are created from the same set of forms (e.g., words and shape) (Bialystok, 2010; Blumenfeld and Marian, 2014). Stimulus–stimulus inhibition is likely to be recruited for bilingual language processing (comprehension and production) and refers to conflict between co-activated language representations (Blumenfeld and Marian, 2014).

The verbal Stroop task was selected as it has previously been associated with bilingual advantages (e.g., Bialystok et al., 2008). It is proposed to examine prepotent response inhibition, which is the ability to suppress dominant, automatic, or prepotent responses (Miyake et al., 2000). It measures verbal cognitive control as the task involves verbal (i.e., semantic and linguistic) elements. It is reasoned that the control processes that are implicated in task performance (i.e., stimulus–stimulus inhibition) are reflective of bilinguals’ linguistic processing and control, in which the conflict between co-activated language representations has to be resolved (Blumenfeld and Marian, 2014). In the verbal Stroop task, the two stimulus dimensions that create cognitive conflict are in the color of the word’s ink (e.g., green font, blue font) and the meaning of the word (e.g., greenness, blueness). In this task, it involves the reading of color words that implicates linguistic demands, and incongruency stems from the semantic properties of the stimuli (i.e., meaning of the word). Linguistic conflict may be argued to arise between the color of the word stimuli (e.g., ink color of word stimuli is blue in color) and meaning of the word (e.g., word stimuli is spelt and read as “green”). Participants have to actively monitor and inhibit the tendency to respond to the meaning of the word (e.g., green, blue), and focus their attention on the color of the word’s ink instead. For all trial types, participants are required to respond according to the color of the font instead of the word, and respond to a designated key. There are three types of trials based on four colors—red, yellow, green, and blue: (a) neutral trials with a color block in one of the four colors, (b) congruent trials with a color word printed in the same color (e.g., “blue” printed in blue font), and (c) incongruent trials with a color word printed in another color (e.g., “red” printed in yellow font). In the incongruent trials, participants have to inhibit the distracting information and focus only on the color of the font, and responses tend to be slower and less accurate.

**FIGURE 1:**
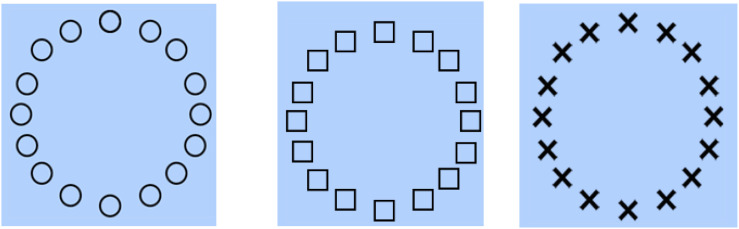
Sample of stimuli used in the trial types of the Global–Local task. The first image is a sample of a congruent trial type. The second image is a sample of an incongruent trial type. The third image is a sample of a neutral trial type.

While a bilingual advantage in verbal processing and control might be expected (e.g., Green, 1998; Green and Wei, 2014), another aspect of bilingual cognitive advantage is that it could extend to non-verbal domains (Bialystok and Shapero, 2005; Bialystok, 2010; Singh and Mishra, 2013). Past evidence has shown a bilingual advantage in visual information processing tasks such as the Flanker task (Hofweber et al., 2020), Simon task (Bialystok et al., 2004), Attentional Network task (Costa et al., 2008, 2009), color–shape task (Hartanto and Yang, 2016), and Global–Local task (Bialystok, 2010). In these non-verbal tasks, the stimulus and conflict are perceptual and spatial in nature (i.e., they are not words, as in the Stroop task). Hence, they tap into non-verbal conflict-resolution skills. For this study, the Global–Local task was used to investigate non-verbal (i.e., perceptual) cognitive control. This task has been used to measure the ability to inhibit attention to salient aspects of perceptual information (Navon, 1977). In the Global–Local task, participants are required to interpret a display of shapes (square or circle) by selectively attending to specific features of the image. In this task, participants are shown a global stimulus (e.g., a shape such as a square or circles) that is constituted from smaller “local” shapes that are either the same as (congruent) or different from (incongruent) the larger shape (note that, in some cases, letters are used, but in the current study, non-linguistic forms are used) (Figure 1). Potential conflict between the two levels is observed in that each is created from similar sets of forms, thus increasing processing demands when the levels are different shapes. It has been found that the global images tend to be processed faster and more accurately than local ones (Bialystok, 2010). Inhibitory control is required to shift between the focus on global or local images, and monitoring and switching demands are further implicated because stimuli can be congruent or incongruent. Participants are tasked to identify either the global or local stimulus, depending on the task condition.

Although the mechanism for performance on the verbal Stroop and Global–Local tasks both invoke the need for inhibitory control, monitoring, and resolution of conflict, differences lie in the nature of the stimulus (verbal vs. non-verbal), which might implicate different domains of cognitive control. Current evidence shows the association between sentential language switching with non-verbal cognitive control (e.g., Hartanto and Yang, 2016; Hofweber et al., 2016, 2020). This study seeks to examine the extent of language switching and its effects on both verbal and non-verbal cognitive control.

To examine the range of cognitive control processes that the ACH incorporates, various outcome measures were employed to tease these processes apart (Table 1). These measures were chosen based on the propositions of the ACH, previous findings that report bilingual associations with these respective cognitive control processes (e.g., Costa et al., 2009; Hilchey and Klein, 2011; Hofweber et al., 2020), and based on past research on adult cognitive control performance (Rubin and Meiran, 2005). This study also attempts to make comparable outcome measures for both the verbal Stroop task and the non-verbal Global–Local task.

Overall, the aim of the current study is to examine the effects of language switching engagements, based on predictions derived from the Adaptive Control Hypothesis framework (Green and Abutalebi, 2013). Although this model is widely referenced, it is observed that there is no study to date that has tested its assumptions and predictions directly or comprehensively. Thus, this study is original in its attempt to do so. This study examines the model’s predicted language and cognitive control effects associated with bilinguals’ engagement in different interactional contexts. Understanding this is significant for testing an influential bilingual language control model. It will bring us a step closer to understanding the nature of the relationship between language switching and cognition, and in linking more precisely the cognitive control processes that are involved in this interaction (see Laine and Lehtonen, 2018 for a review). This study aims to address the following three questions:

*Research Question 1 (RQ1)*: Do bilinguals vary in their engagement in the three interactional contexts described by the ACH?

Based on the Adaptive Control Hypothesis, it is hypothesized that bilingual individuals will differ in the type of interactional contexts in which they primarily engage, differentiating between the pattern of single language context, dual-language context, or dense code-switching context. This claim will be examined using self-reported and observed behavioral measures. Specifically, it is predicted that self-reported interactional contexts will be systematically related to task-induced language switching behaviors (alternating word switching, semi-cued and uncued inter-sentential and intra-sentential switching) (see Table 2). In both the single-language context and dual-language context, language task schemas are proposed to be in a competitive relationship (Green and Abutalebi, 2013). As such, it is hypothesized that frequent engagement in both these contexts, will be associated with more natural production of controlled types of language switches (e.g., inter-sentential switching). This is especially hypothesized for the dual-language context, where bilinguals are proposed to switch between their languages within conversations, but not within utterances (intra-sentential switching). For the dual-language context, it is further hypothesized that language control effects will also be observed in word switching, where language control demand is the highest (alternating language switching) (Declerck and Philipp, 2015). For the dense code-switching context, primary involvement in this context will demonstrate more intra-sentential switching. These predictions are based on the language control outcomes proposed within the Adaptive Control Hypothesis.

**TABLE 2:** Self-report items for measuring individual differences in bilingual interactional contexts and their hypothesized associations with language switching behaviors.

	**Interactional contexts**		
**Measures**	**Single-language context**	**Dual-language context**	**Dense code-switching**
**Self-report items**	⚪ I tend to speak only one language in one environment and another language in another environment ⚪ I tend to speak both languages in the same environment (R) ⚪ I tend to switch languages during a conversation (R)	⚪ I tend to speak both languages in the same environment ⚪ I switch languages between sentences when conversing with others	⚪ I include Chinese words or phrases into English conversations I have with others ⚪ I include English words or phrases into the Chinese conversations I have with others
**Word switching task** (Cued)		+	
**Story Narration task** (semi-cued)—Induced inter-sentential switches	+	+	
**Story Narration task** (semi-cued)—Induced intra-sentential switches			+
**Naturalistic Conversation task** (uncued) Naturally occurring inter-sentential switches	+	+	
**Naturalistic Conversation task** (uncued) Naturally occurring intra-sentential switches			+

*+ indicates that the context is associated with language switching behaviors (word switching, inter-sentential switches, and intra-sentential switching) in different language switching tasks. (R) indicates reverse-coded.*

Alternatively, there may be fluidity in the types of bilingual’s linguistic environments the current sample experiences within a multilingual society. Due to the prevalence of bilinguals and presence of multiple languages, it is likely that the dual-language context would be most dominant in such an environment. However, the dense code-switching context might also be present due to the way that bilinguals may switch intra-sententially between their languages. While it may be that the dual-language context prevails, other contexts (e.g., dense code-switching) might also present. The null hypothesis is that there may not be distinct sets of individuals who have primary engagement in one context, with a predictable set of switching behavior types. Or, even if groups within different primary contexts do exist, there will not be any differences observed between bilinguals of the different contexts in their language switching behaviors.

*Research Question 2 (RQ2):* What is the relationship between bilinguals’ primary engagement in certain interactional contexts with verbal and non-verbal cognitive control?

It is hypothesized that bilinguals’ primary engagement in a dual-language context will demonstrate efficiency across all verbal and non-verbal cognitive control processes. This is based on the Adaptive Control Hypothesis, which specifically predicts that cognitive efficiency will be seen in terms of faster RTs and smaller cognitive costs (advantage) in verbal and non-verbal cognitive control measures across all control processes of goal maintenance, interference control, selective response inhibition, task engagement, and disengagement (Table 1). The null hypothesis is that higher engagement in the dual-language context is not associated with any cognitive control processes.

*Research Question 3 (RQ3):* What is the relationship between bilinguals’ observed language switching behaviors with verbal and non-verbal cognitive control?

It is hypothesized that controlled types of language switches (i.e., alternating word switching and inter-sentential switches) will be associated with greater cognitive control efficiency, whereas less controlled language switching (i.e., intra-sentential switches) will be associated with less cognitive control efficiency (i.e., increased costs). These predictions are based on the view that language switches such as inter-sentential switching involve greater cognitive control due to greater language separation and necessitated control needed to suppress non-target varieties (Muysken, 2000; Green and Abutalebi, 2013; Green and Wei, 2014; Hartanto and Yang, 2016). In view that language switching involves the activation and control of bilinguals’ language representations, this study predicts their engagement with verbal cognitive control processes. The null hypothesis is that there will not be differential effects observed between the prevalence of language switching types and cognitive control efficiency.

## Materials and Methods

### Participants

Seventy-four English–Mandarin young adult bilinguals (*M*_*age*_ = 17.97 years, *SD*_*age*_ = 1.21) were recruited from a tertiary education institution in Singapore. Participants completed a language background questionnaire (LBQ) that asked for details about each of their language histories and language switching practices. Participants in this study were all exposed to two languages (age of bilingual exposure, *M* = 2.54 years, *SD* = 2.12), and started to actively use both languages from an early age [age of active bilingualism (AoAB), *M* = 5.83 years, *SD* = 3.31]. All participants also reported proficiency in both their languages (English and Mandarin) and that they used both languages frequently. Table 3 shows the descriptives of participants’ language background measures.

**TABLE 3:** Descriptives of language background measures.

	***M***	***SD***
Age	17.97	1.21
Age of English exposure	1.79	1.87
Age of Mandarin exposure	1.89	1.80
Age of active bilingualism^a^	5.83	3.31
Average frequency of English use^b^	4.84	0.41
Average frequency of Mandarin use^b^	3.38	1.04
English self-reported proficiency^c^	4.25	0.93
Mandarin self-reported proficiency^c^	3.32	1.03
Engagement in single-language context^d^	8.67	2.60
Engagement in dual-language context^e^	6.03	1.90
Engagement in dense code-switching context^f^	6.61	2.17

**N* = 70–74 for all analyses. ^*a*^Age of active bilingualism is the age at which they reported to actively use the other language aside from the language they first used. ^*b*^Average frequency of language use was attained based on ratings on a 5-point scale for frequency in hearing, speaking, reading, and writing English and Mandarin on a daily basis: 1 = never, 2 = rarely (less than an hour a day), 3 = sometimes (a few hours a day), 4 = often (half a day), 5 = full or almost entire day. ^*c*^Participants rated their proficiency in understanding and speaking using a 5-point scale: 1 = can understand/use simple everyday expressions, 2 = can understand/communicate routine and basic information, 3 = can independently understand/use language to carry on conversation/task, 4 = can independently understand/use language with fluency and spontaneity, 5 = can proficiently and flexibly understand/use language for social, academic, and professional purposes. An average score of their ratings in understanding and speaking were calculated. ^*d*^Participants rated their frequency of engaging in the single-language context on three items, using a 5-point scale of 1 (never) to 5 (always) (Table 2). Higher scores indicate higher engagement in a single-language environments. Scores range from 5 (lowest) to 17 (highest). ^*e*^Participants rated their frequency of engaging in the dual-language context on two items, using a 5-point scale of 1 (never) to 5 (always) (Table 2). Higher scores indicate higher engagement in a dual-language context. Scores range from 5 (lowest) to 10 (highest). ^*f*^Participants rated their frequency of engaging in the dense code-switching context on two items, using a 5-point scale of 1 (never) to 5 (always). A higher score indicates higher engagement in a dense code-switching context. Scores range from 5 (lowest) to 10 (highest).*

### Materials

#### Language Proficiency

The semantic verbal fluency task was administered in both English and Mandarin (Van Assche et al., 2013). The categories for both English and Mandarin tasks were Animals and Kitchen items. Participants were instructed to verbally list as many words as they could, within 1 min, for each category in each language. Participants were scored based on the number of correct words produced for category.

Performance on English and Mandarin verbal fluency was also used as a measure of bilinguals’ relative balanced proficiency. From the total number of correct words produced in English and Mandarin, *z*-scores for each language were attained across all participants (i.e., one *z*-score for English, one *z*-score for Mandarin). Thereafter, a difference score between the English *z*-score and Mandarin *z*-score was calculated as an indicator of bilinguals’ individual relative balanced proficiency (see Yow and Li, 2015, for measures of balanced bilingualism). A score of 0 indicates relative balanced proficiency in the two languages.

#### Word Switching (Alternating Language Switching)

Word switching was based on the semantic verbal fluency task and adapted from the verbal task switching measure in Yim and Bialystok (2012). In this task, all participants were given the category of vegetables and were required to continue generating as many words in this category for 1 min, by alternating between languages without repetition of the same words for each language. Participants were instructed that they could start with whichever language they wanted to (i.e., English or Mandarin). For example, if the participant’s starting language was English, the participant may respond with *spinach* in English, and *lettuce* in Mandarin, and so forth. Task instructions were given in both English and Mandarin. Participants’ responses were recorded with an audio recording device. Raw scores are participants’ correct responses for words of each language and total number of correct words. The task constraints on this task involved the strongest level of cued switching, as it was a requirement for successfully performing the task, and only correct switches were included in the scores.

#### Sentential Switching

Utterance and sentential-level switching was assessed through two tasks. The first task was a recount of a story and the other was a naturalistic conversation in which participants discussed their favorite childhood stories with the experimenter. These tasks were designed to combine characteristics of controlled and naturalistic language switching behaviors. Both differ in their degree of imposed control in using both languages, with the story recount task considered as semi-cued switching, and the conversation task considered as uncued switching. For both tasks, English, Mandarin, inter-sentential switches, and mixed utterances (intra-sentential switches) were counted.

##### Story recount task (semi-cued language switching)

This task was self-designed and is an adaptation of the recounting task used by Toribio (2001). This task, which was designed with the intention of engaging the participants in bilingual speech production, measures language switching performance through a monological narration of a familiar fairy tale story. In this task, participants first listened to a short verbal narration of an audio recording to the introduction of the story, *The Little Red Riding Hood*. The story was narrated along with an auto-played sequence of cartoon picture cards (with no subtitles or words) depicting the story through PowerPoint slides. The verbal narration comprised eight sentences, which was a combination of English-only (two sentences), Mandarin-only (two sentences), and English–Mandarin mixed sentences (i.e., intra-sentential switching) (two sentences). Inter-sentential switching was incorporated twice within the narration. Participants were then instructed in both English and Mandarin, to continue to recount the rest of the story based on all the picture cards provided in the slides. They were also told that they had to use both English and Mandarin in their recount and could do so in a way that was natural to how they would normally use both their languages. They were encouraged to tell the rest of the story as descriptively and as detailed as possible using both languages. There was no time limit and the task ended when they completed all the picture cards.

##### Naturalistic conversation task (uncued language switching)

Language switching was also assessed through a semi-structured conversation in which participants discussed with an English–Mandarin bilingual experimenter, on the topic of childhood stories. As this task followed from the Story Narration task, experimenters asked questions related to what they thought about the story of *The Little Red Riding Hood*, what their favorite childhood story was, and what they liked about that story. To maximize language switching in an artificial laboratory setting within an English-dominant context, all questions (even follow-up questions) were communicated in Mandarin. This was done purposefully based on findings and feedback from an earlier pilot of the task. To ensure that there was sufficient conversational exchange, experimenters were trained to maintain the conversation through naturalistic and elaborative questions, and had to engage in conversation with each participant for at least 5 min.

#### Language Background Questionnaire

A self-report language background questionnaire was used to examine bilinguals’ language proficiency in both languages, AoAB, and types of language switching behaviors. The questionnaire was adapted from Lim et al. (2008) Determining Language Dominance in English–Mandarin Bilinguals questionnaire, Marian et al. (2007) The Language Experience and Proficiency Questionnaire (LEAP-Q), and Rodriguez-Fornells et al. (2012) Bilingual Switching Questionnaire (BSWQ). Questions asking participants about the ages they were first exposed to each of the languages were used to ascertain the AoAB. To obtain a measure of language proficiency measures, participants had to indicate their perceived level of proficiency in each of the languages based on a 5-point scale. To measure language switching, using a 5-point scale, participants reported on their frequency of engagement in various language switching contexts (single-language context, dual-language context, and dense code-switching context) and behaviors (e.g., inter-sentential and intra-sentential switching).

#### Verbal Stroop Task

A computerized version of the Stroop (1935) color-naming task was used to measure verbal cognitive control processes (interference control of pre-potent tendencies). There were four types of trials based on four colors—red, yellow, green, and blue: (a) baseline trials with a color-word presented to assess baseline reading, (b) neutral trials with a square filled in one of the four colors, (c) congruent trials with a color word printed in the same color (e.g., yellow printed in yellow), and (d) incongruent trials with a color word printed in a different color (e.g., yellow printed in blue). All words were displayed in 36-point Arial font and all letters were in lowercase. Participants viewed it at a distance of 40 cm.

For all trials, participants were instructed to respond according to the color of the font by pressing a designated key on the keyboard (S, F, H, and K for red, yellow, green, and blue, respectively). The keys were marked with matching stickers indicating the first letter of the color (R, Y, G, and B). Each trial began with a centered black fixation cross (+) presented against a white background for 500 ms, followed by the stimulus that remained on the screen for 4000 ms or until a response was made.

Sixteen practice trials (four trials for each trial type) were presented to the participants. A total of 144 test trials were presented after the practice trials. All participants completed the first block consisting of the baseline color–word reading trial, and the second block required participants to indicate the color of the square. They then proceeded with a block of 24 congruent trials, and a block of 24 incongruent trials. The test order of these two blocks was counterbalanced across participants. The last block was a mixed block of 48 trials with an equal number of congruent and incongruent trials. The order of the trials within each block was randomized for each participant. To measure various verbal cognitive control processes aligned with the Adaptive Control Hypothesis (goal maintenance, interference control, selective response inhibition, and task engagement and disengagement), respective scores and corresponding measures were used to reflect performance in these various processes (see Table 1).

#### Non-verbal Global–Local Task

In this study, this task was based on a design developed by Andres and Fernandes (2006), and adapted from the Global–Local task by Bialystok (2010). This task is purported to assess the dominance of attending to global configurations than compositional detail in perceiving spatial patterns (Navon, 1977). This non-verbal task requires perceptual processing of the overall and component features of a complex stimulus, and potential conflict between two levels are introduced, in that both global and local images are created from the same set of forms (Figure 1). Depending on the task rules, participants have to shift their locus of attention between global and local images, while inhibiting the perceptual conflict at the same time. Thus, this task requires non-verbal cognitive control processes related to monitoring, interference control, and engagement/disengagement. To align with the Adaptive Control Hypothesis, respective scores and corresponding measures were also used to reflect performance across these various cognitive control processes (see Table 1).

Each trial began with a black fixation cross (+) presented in the center of the screen against a blue or yellow background for 500 ms, followed by the stimulus that remained on the screen for 4000 ms or until a response was made. The stimuli were approximately 6 cm high and wide. There were two tasks, each based on a different type of stimulus. Participants were required to identify either global or local shapes based on the cue indicated by the color of the background of the trial. If they were required to identify the global shape, a blue background would be shown. If they were required to identify the local shape, a yellow background would be shown. The stimuli were circles or squares (or Xs for neutral). Participants indicated the identity of the relevant stimulus by pressing designated keys. Each response key was assigned to one of the two stimuli (press Z for circle and M for square). The keys were marked with matching stickers indicating the first letter of the shape (C, S).

Instructions were presented at the start of each block explaining whether the global or local shapes were targeted. The neutral stimuli were never a response option; for example, a local X composed of local circles only had a response key associated with the circles. Twelve practice trials (two trials for each trial type) were presented to the participants. There were a total of three types of experimental blocks: global shapes, local shapes, and mixed global and local shapes. In each of the global and local blocks, there was a total of 42 trials (14 trials for congruent, incongruent, and neutral trials), while the mixed block consisted of 56 trials (14 trials for global congruent, global incongruent, local congruent, and local incongruent). This yielded a total of 140 experimental trials across three blocks. The test order of these two blocks was counterbalanced across participants. The last block was a mixed block of 48 trials with an equal number of congruent and incongruent trials. The order of the trials within each block was randomized for each participant.

#### Measures of Cognitive Control Processes

This study selected and defined measures to align closely with the cognitive control processes proposed in the Adaptive Control Hypothesis (Table 1). Note that the Stroop task is considered to reflect verbal cognitive control while the Global–Local task reflects non-verbal cognitive control. Equivalent outcome measures were made for both verbal and non-verbal tasks. These measures were chosen based on (1) description of control processes discussed within the Adaptive Control Hypothesis, (2) earlier evidences that have found bilingual associations with these respective cognitive control processes (e.g., Costa et al., 2009; Hilchey and Klein, 2011; Blumenfeld and Marian, 2014; Hofweber et al., 2016, 2020), and (3) past research on adult cognitive control performance (Rubin and Meiran, 2005). These verbal and non-verbal measures of goal maintenance, interference control, selective response inhibition, and task engagement and disengagement, were used as dependent measures for analyses in this study.

For each task, more difficult trial types (i.e., incongruent trials) and conditions (i.e., mixed block) were selected for analysis. For the verbal Stroop task, incongruent trials (as compared to congruent trials) are proposed to necessitate more cognitive control and tend to be processed slower and less accurately (Stroop, 1935; Blumenfeld and Marian, 2014). For incongruent trials, there is a need to inhibit the pre-potent tendency to respond to the meaning of the word (e.g., green, blue) and focus their attention on the color of the word’s ink instead. Accordingly, incongruent trial types were selected for analysis. For the non-verbal Global–Local task, the usual finding is that local trials are purported to be more difficult than global trials as they tend to be processed slower and less accurately due to the natural perceptual inclination to identify and process global rather than local images (Bialystok, 2010). Within this task, incongruent trial types are also more difficult due to the perceptual conflict that is presented in the image. For incongruent trial types, the global stimulus (e.g., a shape of a square) is constituted from smaller “local” shapes that are different from (incongruent) the larger shape (e.g., a shape of a circle). Thus, local incongruent trial types were selected for analysis.

As a measure of verbal and non-verbal goal maintenance, a mixing cost was used. Mixing cost is argued to reflect global and sustained cognitive control processes (Braver et al., 2003; Rubin and Meiran, 2005). For the verbal Stroop task (1935), mixing cost was measured as the RT difference between incongruent trials in the mixed and pure blocks. For the Global–Local task, mixing cost was measured through difference in RTs between local incongruent trials in the mixed and pure blocks.

To measure verbal and non-verbal interference control: conflict monitoring, overall RTs for incongruent trials (mixed block) was used for the Stroop task, and overall RTs for local incongruent trials (mixed block) was used for the Global–Local task. This follows from Costa et al. (2009), who found bilingual advantages in conflict monitoring as seen through faster overall RTs in both congruent and incongruent trial types in the mixed block (mixed block is the most difficult condition). In this study, only incongruent trial types are examined. To measure verbal and non-verbal interference control: interference suppression, the Stroop effect was used for the verbal Stroop task, while the conflict effect was used for the non-verbal Global–Local task. The Stroop effect is the RT difference between incongruent trial types (pure block) and congruent (neutral) trial types (pure block) (Yow and Li, 2015; Wright, 2017). The conflict effect is the RT difference between incongruent local trials (pure block) and congruent (neutral) local trials (pure block) (Hofweber et al., 2020).

Selective response inhibition is proposed to reflect the control ability to suppress or inhibit an automized motor response (Booth et al., 2003; Hofweber et al., 2020). In this study, to measure verbal selective response inhibition, overall RTs on incongruent trials (pure block) in the Stroop task were used. To measure it non-verbally, overall RTs on local incongruent trials (pure block) were used. Lastly, to measure non-verbal task engagement and disengagement, the switching cost was used for both the verbal Stroop task and non-verbal Global–Local task. The switching cost is argued to reflect more transient cognitive control processes (Braver et al., 2003; Rubin and Meiran, 2005). In both tasks, RT differences between switch and repeat trials within the mixed block were taken.

### Procedure

This study was approved by the institution’s Institutional Review Board. All participants provided informed consent before participating. Before the session, participants had to complete the Language Background Questionnaire via an e-survey platform. They were then scheduled a face-to-face session in which cognitive tasks and language tasks were administered. Trained research assistants, who are all English–Mandarin bilinguals, administered the tasks individually to participants. All participants completed the tasks across one session lasting about 1 h. For all language tasks, each participants’ responses and utterances were audio-recorded and transcribed afterward. For cognitive control measures, trial accuracies and RTs were recorded using Superlab (version 5).

Participants would start their session with either language tasks or cognitive tasks, and this was counterbalanced across all participants. For participants who started with language tasks, they would begin with a verbal fluency task in one language (e.g., English) followed immediately with the other language (e.g., Mandarin). The order of languages to be assessed was counterbalanced as well. For each language, the category of words was counterbalanced (animals and kitchen items). Thereafter, they would complete the word switching task, where they have to switch in producing words in the category of “vegetable.” After that, they would complete the Story Narration and Naturalistic Conversation. Participants would then end their session with the two executive control tasks (Stroop task and Global–Local task), which were counterbalanced across all participants as well.

For participants who started the session with the cognitive tasks, they would begin with either the Stroop or Global–Local task, and this was counterbalanced across participants. After the completion of the cognitive tasks, they would complete the language tasks where they would start with a verbal fluency task in one language (e.g., Mandarin) followed by the other (e.g., English). They then completed the word switching task followed by the Story Narration task and ended their session with the Naturalistic Conversation.

## Data Preparation

### Transcription of Language Switches

Participants’ utterances during the Story Narration task and Naturalistic Conversation sessions were transcribed in accordance with CHAT and the transcriptions were analyzed using CLAN (MacWhinney, 2000). A separate team of research assistants who were native language speakers of English and Mandarin were involved in the transcription and checking process. Individuals in this team were not involved in data collection and task administration, and so they were blind to the conditions of the study. The research assistants independently transcribed the audio recordings assigned to them. In accordance with the transcription and reliability checking methods (Lust and Blume, 2016), another research assistant checked through each transcription for errors or missing data. All transcriptions were checked sentence by sentence, and any discrepancies were verified and discussed before any changes were made.

In all transcriptions, onomatopoeia (imitation of sounds, e.g., animal sounds) and ambiguous communication in both languages (e.g., uh, ah, oh) were excluded from all analyses. SCE (Singapore Colloquial English) is a commonly spoken form of English in Singapore. As such, SCE particles (e.g., meh, la, leh, see Rubdy, 2007) and words that were not English or Mandarin (e.g., “simi,” a Hokkien word which means “what”) were all marked as non-words and excluded from the analyses. Following from Yow et al. (2018), the basic unit of analysis is an utterance, which is defined as “a word or group of words with a single intonation contour” (Lanza, 1992). A pure utterance in either English or Mandarin, consisting of a string of words only in one language, carries a singular idea and excludes intra-sentential switches and utterances that contain translations and imitations of other languages. Mixed utterances are those in which both languages are included in the same utterance (Table 4).

**TABLE 4:** Example of language switching types.

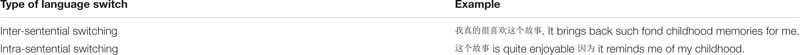

#### Types of Sentential Switches

Inter-sentential and intra-sentential switching were coded from participant’s utterances. Each type was operationally defined using Muysken (2000) classification of three code-switching types, which differ in their language separation and co-activation (see Treffers-Daller, 2009; Green and Wei, 2014; for review). In *alternation*, bilinguals switch between their languages between turns or utterances. This involves producing structurally equivalent stretches of two languages. In *insertion*, lexical items from one language is inserted into the language structure of another language. In *congruent lexicalization*, the lexical and grammar structure of both languages are shared and co-activated. In this study, inter-sentential switching follows that of alternation, while intra-sentential switching would follow that of insertion and congruent lexicalization due to the prevalence of English-based creole in Singapore, where the difference between insertion and congruent lexicalization is not clearly separable (see Hartanto and Yang, 2016). The percentage of intra-sentential utterances made by each participant was obtained by dividing the number of cases by the total number of utterances spoken by each participant. The percentage of inter-sentential utterances was derived based on the total number of times that each participant switched from one full utterance in one language (e.g., English) to another language (e.g., Mandarin), and dividing it by the total number of utterances spoken.

### Classification of Bilinguals in the Three Interactional Contexts

To measure bilinguals’ self-reported engagement in the three types of interactional context, six survey items were selected from the Language Switching Questionnaire (Table 2). Pearson correlational analyses were performed on the six items (Table 5). Along with theoretical predictions, findings from this correlational analysis were also used as a guide to determine items for each interactional context. For each interactional context, items that aim to measure each type of interactional context were selected with other relevant items that suggest significant and strong correlations. The frequency of engagement in the Single-Language Context is measured through three items. These items measure the extent to which bilinguals keep their languages apart, the extent to which they use both languages in the same environment (reverse coded), and their engagement in general switching (reverse coded) (maximum score is 17). Dual-Language Context is measured through two items. These two items measure the frequency with which both languages are spoken in the same environment, and the frequency of switching between sentences when conversing with others (i.e., intersentential switching) (maximum score is 10). Dense code-switching is measured through two items. These two items measure the frequency with which bilinguals include words and phrases from one language (e.g., Chinese or English) into the other when they converse with others (maximum score is 10). A composite score for each interactional context was taken as a measure of bilinguals’ reported engagement in each of the three contexts. Each participant had three scores, one for each interactional context.

**TABLE 5:** Correlations between self-reported language switching items.

	**1**	**2**	**3**	**4**	**5**	**6**
I tend to speak only one language in one environment and another language in another environment (SLC)	1					
I tend to speak both languages in the same environment (DLC)	−0.29*	1				
I switch languages between sentences when conversing with others (inter-sentential)	–0.17	0.47**	1			
I tend to switch languages during a conversation (general switching)	−0.24*	0.56**	0.70**	1		
I include Chinese words or phrases into English conversations I have with others (intra-sentential)	–0.17	0.42**	0.58**	0.61**	1	
I include English words or phrases into the Chinese conversations I have with others (intra-sentential)	–0.02	0.41**	0.55**	0.62**	0.68**	1

*N* = 70–74 for all analyses. **p = 0.01 (two-tailed), *p = 0.05 (two-tailed).

## Results

### Language Measures

Demographic information and mean scores on the self-reported language background measures are presented in Table 3. Participants reported on their language history, their proficiency in their languages, usage of both languages, and language switching behaviors. Objective language proficiency and measures included performance on the verbal fluency tasks for each language, the word switching task, and linguistic performance on the Story Narration and Naturalistic Conversation task. Descriptives of objective language measures are presented in Table 6.

**TABLE 6:** Descriptives of objective language proficiency measures and language switching behaviors.

	***M***	***SD***
English verbal fluency	39.05	10.49
Mandarin verbal fluency	21.89	9.31
Word switching task	6.91	2.93
**Story narration task**		
• Total number of utterances	28.62	13.01
• English utterances (%)	32.16	15.81
• Mandarin utterances (%)	28.46	16.03
• Inter-sentential switches (%)^a^	18.09	14.47
• Intra-sentential switches (%)^b^	39.37	23.20
**Naturalistic conversation task**		
• Total number of utterances	19.33	14.68
• English utterances (%)	29.45	33.27
• Mandarin utterances (%)	54.63	34.63
• Inter-sentential switches (%)^a^	8.18	5.93
• Intra-sentential switches (%)^b^	18.36	20.68

**N* = 70–74 for all analyses. The total number of all utterances is the sum of pure English, pure Mandarin, and intra- and inter-sentential utterances. Inter-sentential switch utterances comprise of only pure utterances. Intra-sentential switches are mixed language utterances. ^*a*^The percentage of inter-sentential utterances was derived based on the total number of times that each participant switched from one full utterance in one language (e.g., English) to another language (e.g., Mandarin), and dividing it by the total number of utterances spoken. ^*b*^The percentage of intra-sentential utterances made by each participant was obtained by dividing the number of cases by the total number of utterances spoken by each participant.*

### Data Trimming for Cognitive Control Measures

RT analyses were based only on trials with correct responses. Firstly, to attain individual task accuracy, trials with incorrect responses were omitted. Thereafter, trials with RTs below 200 ms or above 2.5 SDs from the mean in each condition were trimmed for each individual participant. This allows for the best measure of central tendency for each condition (Friedman et al., 2011; Yow and Li, 2015). Data were further trimmed across the entire sample for each condition, in which trials with accuracy and RTs below 2.5 SDs from the overall mean of each condition were omitted. All of this resulted in the exclusion of 1% of trials from the Stroop task and 3% of trials from the Global–Local task. No participants were removed for performance reasons in any of the two tasks. Table 7 shows descriptives for RTs of all cognitive tasks and measures.

**TABLE 7:** Descriptives of RTs for cognitive task measures (RTs across trial types and conditions).

	***M***	***SD***
**Goal monitoring**		
• Verbal Stroop (mixing cost)	92.05	267.67
• Non-verbal Global–Local (mixing cost)	784.49	359.83
**Interference control: Conflict monitoring**		
Verbal conflict monitoring		
• Incongruent trials (mixed block)	1123.01	259.88
Non-verbal conflict monitoring		
• Local incongruent trials (mixed block)	1486.87	400.75
**Interference control: Interference suppression**		
• Verbal Stroop (Stroop effect)	194.96	247.95
• Non-verbal Global–Local (conflict effect)	42.50	180.15
**Selective response inhibition**		
• Verbal Stroop (incongruent RTs pure block)	1032.10	232.31
Non-verbal		
• Local incongruent (pure block)	702.38	169.34
**Task engagement/disengagement**		
• Verbal Stroop (switch cost)	34.87	118.96
• Non-verbal GL (switch cost)	87.64	97.73

*Refer to Table 1 for verbal and non-verbal cognitive control task measures for the Stroop task and Global–Local task.*

### RQ1—Correlations Between Bilinguals’ Engagements in the Three Interactional Contexts and Naturalistic Language Switching Behaviors

To examine the first research question, Pearson correlations were first run between six items measuring self-reported engagement in language switching behaviors of the three interactional contexts (Table 5). Significant negative correlations were observed between the item measuring single-language context switching with the item measuring dual-language context switching (*r* = −0.29, *p* < 0.05) and general language switching (*r* = −0.24, *p* < 0.05). This suggests that bilinguals’ higher frequency of engagement in an interactional context in which languages are used in separate environments (single-language context) is reversely related to engagement in an interactional context in which both languages are used in the same environments (dual-language context) and in the engagement of language switching behaviors during conversations (general switching). However, the small negative correlations indicate that within the current sample, there is not a strong distinction between bilinguals who engage primarily in a single-language context or in a dual-language context and in language switching behaviors. This could suggest fluidity between bilinguals’ engagement in the single-language and the dual-language context.

Significant positive correlations were observed between the item measuring the dual-language context with inter-sentential switching (*r* = 0.47, *p* < 0.01), with general switching (*r* = 0.56, *p* < 0.01), and with the two items measuring intra-sentential switching (i.e., dense code-switching contexts) (*r* = 0.41; *r* = 0.42, both *p*s < 0.01) (Table 5). This suggests that higher frequency of engagement in an interactional context in which both languages are used in the same environment is associated with higher frequency of engagement in general language switching, inter-sentential switching, and intra-sentential switching (dense code-switching context). These correlations of moderate strength suggest that a less clear division exists between individuals engaging primarily in a dual-language context or dense code-switching context, and there is substantial fluidity between these two contexts. Overall, the fluidity of bilinguals’ engagement in the three interactional contexts are observed. Correlations between bilinguals’ reported engagement in the three interactional contexts are presented in Table 8.

**TABLE 8:** Correlations between bilinguals’ reported engagement in the three interactional contexts.

	**1**	**2**	**3**
Single-language context	1		
Dual-language context	−0.80**	1	
Dense code-switching context	−0.53**	0.62**	1

*N* = 70–74 for all analyses. **p = 0.01 (two-tailed), *p = 0.05 (two-tailed).

To examine the relation of self-reported engagement in each interactional context and predicted language switching behaviors, three sets of Pearson correlations were run between each context (single-language context, dual-language context, and dense code-switching context) with observed verbal language switching behaviors (alternating word switching, and semi-cued and un-cued inter-sentential and intra-sentential switches) across language switching tasks (Story Narration task and Naturalistic Conversation task) (refer to the Section “Classification of Bilinguals in the Three Interactional Contexts” for classification of the three interactional contexts). There were no significant correlations observed between different interactional contexts with predicted language switching behaviors (Table 9). These correlations do not support the expected pattern of association between primary engagement in each interactional context and observed language switching behaviors. This suggest that within a multilingual society, bilinguals’ self-reported primary engagement in the different interactional contexts does not associate with a particular expected pattern of dual-language use, with regard to their observed language switching behaviors. Refer to Table 10 for additional Pearson correlation analyses between observed language switching behaviors, Table 11 for correlations between interactional contexts and cognitive control, and Table 12 for correlations between language switching behaviors and cognitive control.

**TABLE 9:** Correlations between reported engagement in interactional contexts with observed language switching behaviors.

	**Word-switching**	**SN: Inter-sentential switches**	**SN: Intra-sentential switches**	**NC: Inter-sentential switches**	**NC: Intra-sentential switches**
Single-language context	–0.14	–0.01	–0.15	0.10	–0.10
Dual-language context	0.11	0.07	0.04	–0.08	0.07
Dense code-switching	0.08	0.09	0.07	0.03	–0.00

*N* = 70–74 for all analyses. **p < 0.01 (two-tailed), *p < 0.05 (two-tailed). SN, Story Narration task; NC, Naturalistic Conversation task.

**TABLE 10:** Correlations between observed language switching behaviors.

	**1**	**2**	**3**	**4**	**5**
Word switching	1				
SN inter-sentential switching	0.01	1			
SN intra-sentential switching	0.05	−0.75**	1		
NC inter-sentential switching	–0.07	–0.14	0.14	1	
NC intra-sentential switching	−0.23*	–0.13	0.22	0.04	1

*N* = 70–74 for all analyses. *p < 0.05 (two-tailed) **p < 0.01 (two-tailed). SN, Story Narration task; NC, Naturalistic Conversation task.

**TABLE 11:** Correlations between interactional contexts with cognitive control measures.

	**Goal maintenance**		**Interference control: Conflict monitoring**		**Interference control: Interference suppression**		**Selective response inhibition**		**Engagement and disengagement**	
	**V**	**NV**	**V**	**NV**	**V**	**NV**	**V**	**NV**	**V**	**NV**
Single-language context	0.18	0.01	0.30*	–0.03	–0.01	0.11	0.12	–0.10	0.27*	–0.02
Dual-language context	–0.15	–0.07	−0.26*	–0.08	–0.09	–0.17	–0.11	–0.04	–0.23	–0.11
Dense code-switching context	–0.12	–0.19	–0.15	–0.13	0.08	–0.19	–0.02	0.10	–0.17	–0.06

*N* = 70–74 for all analyses. **p < 0.01 (two-tailed), *p < 0.05 (two-tailed). **p < 0.01, *p < 0.05. Note: V refers to verbal Stroop task. NV refers to non-verbal Global–Local task.

**TABLE 12:** Correlations between language switching behaviors with cognitive control measures.

	**Goal maintenance**		**Interference control: Conflict monitoring**		**Interference control: Interference suppression**		**Selective response inhibition**		**Engagement and disengagement**	
	
	**V**	**NV**	**V**	**NV**	**V**	**NV**	**V**	**NV**	**V**	**NV**
Word switching	0.04	0.08	–0.22	–0.01	–0.11	–0.14	−0.29*	–0.18	–0.07	–0.16
SN: Inter-sentential switching	0.07	–0.06	–0.08	0.01	–0.18	0.11	–0.16	0.13	–0.14	–0.06
SN: Intra-sentential switching	–0.08	–0.09	0.13	0.08	0.14	–0.10	0.22	–0.01	0.23*	0.15
NC: Inter-sentential switching	0.17	−0.28*	0.30*	−0.28*	0.06	0.06	0.12	–0.07	0.13	–0.05
NC: Intra-sentential switching	0.04	–0.06	0.03	–0.03	–0.07	–0.02	–0.02	0.06	–0.02	0.10

*N* = 70–74 for all analyses. **p < 0.01, *p < 0.05. V, verbal Stroop task; NV, non-verbal Global–Local task; SN, Story Narration task; NC, Naturalistic Conversation task.

### RQ2—Regression Analyses of Engagement in Interactional Contexts With Verbal and Non-verbal Cognitive Control

To address question 2, the relative influence of bilinguals’ primary engagement in different interactional contexts on all verbal and non-verbal cognitive control measures were examined with separate multiple hierarchical regression analyses. Prior to conducting hierarchical multiple regressions, the relevant assumptions were tested. The dual-language context was used as a predictor on all verbal and non-verbal cognitive control measures. It was used because this interactional context is expected to be most highly related to cognitive control processes as predicted within the Adaptive Control Hypothesis (Green and Abutalebi, 2013) (Table 1). It was also used due to the significant correlations between the three interactional contexts (see Table 8).

All collinearity statistics (i.e., Tolerance and VIF) were within accepted limits, and thus, the assumption of multicollinearity was addressed (Hair et al., 1998). The sample size of 74 was deemed adequate given the number of variables to be included in the analysis (Tabachnick et al., 2007). In the first step, age was entered as a control variable. Next, balanced bilingual proficiency was entered as the second step. This was to account for bilinguals’ relative balanced proficiency in both their languages. Bilingual proficiency has been proposed to influence cognitive control processes (e.g., Singh and Mishra, 2012, 2013; Yow and Li, 2015; Xie, 2018). Finally, engagement in dual-language context was entered as the third step as it reflects bilinguals’ degree of dual-language exposure and use of both languages in their linguistic environment. It was entered as a third step to see if controlled language switching within a dual-language context would better explain cognitive control over and above bilingual proficiency alone.

The regression model for the verbal Stroop task (switching cost) was significant (*p* = 0.05). The addition of dual-language context engagement into the third model demonstrated a trend toward improving the overall model and the change in *R*^2^ was approaching significance (*R*^2^ change = 0.05, *F* change = 3.49, *p* = 0.06). Reported engagement in the dual-language context demonstrated a trend toward contributing unique variance to verbal switch costs (β = −0.22, *p* = 0.06) (Table 13). This could suggest that higher engagement in a dual-language context was somewhat predictive of efficiency in verbal task engagement and disengagement (lower switch costs).

**TABLE 13:** Results of hierarchical regression analysis of engagement in dual-language context on verbal task engagement and disengagement.

	**Step 1**			**Step 2**			**Step 3**		
	***B***	***SEB***	**β**	***B***	***SEB***	**β**	***B***	***SEB***	**β**
**Step 1: Age**	7.40	11.90	0.08	10.15	11.64	0.10	12.50	11.50	0.13
**Step 2: Bilingual proficiency**				24.33	12.21	0.24	22.36	12.03	0.22
**Step 3: DLC**							−13.63	7.29	−0.22*
*R*^2^	0.00			0.06			0.11		
Δ*R*^2^				0.06			0.05		
Δ*F*				3.98			3.49		
Overall model significant	0.53			0.12			0.05*		

**p < 0.01, *p < 0.05. This cognitive control measure is the switching cost in the Stroop task (see Table 1). DLC refers to engagement in the dual-language context. The addition of DLC improved the model significantly. DLC demonstrated a trend toward significance (*p* = 0.06).

All other hierarchical regression models were non-significant (*p*s > 0.05). This demonstrates that the degree of bilinguals’ engagement in a dual-language context was not predictive of other cognitive control processes. This is in contrast to the expected predictions between dual-language context engagement and verbal and non-verbal cognitive control processes.

### RQ3—Regression Analyses of Naturalistic Language Switching Behaviors on Verbal and Non-verbal Cognitive Control

To examine research question 3, the influence of bilinguals’ naturalistic production of language switching behaviors on verbal and non-verbal cognitive control processes were examined through separate multiple hierarchical regression analyses. Regression analyses were performed on verbal and non-verbal cognitive control processes with predictors of different language switching behaviors: word switching, inter-sentential, and intra-sentential language switches. As in Section “RQ2—Regression Analyses of Engagement in Interactional Contexts With Verbal and Non-verbal Cognitive Control,” four measures of cognitive control were examined in separate models, for the verbal (Stroop) and non-verbal (Global–Local) tasks.

The relevant assumptions were similarly tested and all collinearity statistics (i.e., Tolerance and VIF) were within accepted limits (Hair et al., 1998). In the first step, age was entered as a control variable. Next, balanced bilingual proficiency was entered as the second step. In the third step, either word switching or sentential language switches (inter-sentential and intra-sentential switching) in the Story Narration task and Naturalistic Conversation task were entered as the third step. Models for goal maintenance, conflict monitoring, response inhibition, and engagement/disengagement are reported for each switching type below.

#### Regression Analysis of Word-Switching (Alternating Language Switching) on Verbal and Non-verbal Cognitive Control

All hierarchical regression models for word-switching on verbal and non-verbal cognitive control processes were non-significant (all *p*s > 0.05). This demonstrates that word-switching performance was not predictive of all verbal and non-verbal cognitive control processes.

#### Regression Analyses of Language Switching in the Story Narration Task (Semi-Cued Switching) on Verbal and Non-verbal Cognitive Control

All hierarchical regression models for inter-sentential switching and intra-sentential switching in the Story Narration task on cognitive control processes were non-significant (all *p*s > 0.05). This demonstrates that bilinguals’ language switching behaviors (inter-sentential switching and intra-sentential switching) in the Story Narration task was not predictive of verbal and non-verbal cognitive control processes.

#### Regression Analyses of Language Switching in the Naturalistic Conversation Task (Uncued Switching) on Verbal and Non-verbal Cognitive Control

Hierarchical regression models of cognitive control processes with predictors of inter-sentential switching and intra-sentential switching in the Conversation task were conducted for each of the four cognitive control processes. The regression model for non-verbal goal maintenance (Global–Local Task: Mixing Cost of Local Incongruent trials) was significant (*p* = 0.05). The addition of inter-sentential switching into the third model significantly improved the overall model and the change in *R*^2^ was significant (*R*^2^ change = 0.12, *F* change = 4.30, *p* = 0.02). Inter-sentential switching contributed significantly to the model (β = −0.31, *p* = 0.01) (Table 14). This suggests that higher production of verbal inter-sentential switches in a naturalistic conversation was predictive of non-verbal goal monitoring efficiency (lower mixing cost).

**TABLE 14:** Regression analysis of sentential switching on non-verbal goal maintenance.

	**Step 1**			**Step 2**			**Step 3**		
	***B***	***SEB***	**β**	***B***	***SEB***	**β**	***B***	***SEB***	**β**
**Step 1: Age**	–16.96	37.05	−0.06	−9.69	37.90	−0.03	−27.02	36.87	−0.09
**Step 2: Bilingual proficiency**				37.73	40.43	0.12	44.37	39.43	0.14
**Step 3: Language switching**									
Inter-sentential switches							−18.69**	7.10	−0.31**
Intra-sentential switches							−2.89	2.35	−0.15
*R*^2^	0.00			0.02			0.13		
Δ*R*^2^				0.01			0.12		
Δ*F*				0.87			4.29*		
Overall model significant	0.65			0.59			0.05*		

**p < 0.01, *p < 0.05. This cognitive control measure is the mixing cost of local incongruent trials from the Global–Local task (see Table 1).

The regression model for non-verbal interference control: conflict monitoring (Global–Local Task: Overall RTs of incongruent trials in mixed block) was significant (*p* = 0.05). The addition of inter-sentential switching into the third model significantly improved the overall model and the change in *R*^2^ was significant (*R*^2^ change = 0.10, *F* change = 3.63, *p* = 0.03). Inter-sentential switching contributed significantly to the model (β = −0.29, *p* = 0.02) (Table 15). This suggests that higher production of verbal inter-sentential switches in a naturalistic conversation was predictive of non-verbal conflict monitoring efficiency (faster RTs).

**TABLE 15:** Regression analysis of sentential switching on non-verbal interference control: conflict monitoring.

	**Step 1**			**Step 2**			**Step 3**		
	***B***	***SEB***	**β**	***B***	***SEB***	**β**	***B***	***SEB***	**β**
**Step 1: Age**	–56.93	41.22	−0.17	–49.96	42.23	–0.15	–66.75	41.47	–0.20
**Step 2: Bilingual proficiency**				36.12	45.05	0.10	40.96	44.35	0.11
**Step 3: Language switching**									
Inter-sentential switches							−20.06*	7.99	−2.51*
Intra-sentential switches							–2.44	2.65	–0.11
*R*^2^	0.03			0.04			0.14		
Δ*R*^2^				0.01			0.10		
Δ*F*				0.64			3.63*		
Overall model significant	0.17			0.29			0.05*		

**p < 0.01, *p < 0.05. This cognitive control measure is the overall reaction times (RTs) of local incongruent trials in the mixed block from the Global–Local task (see Table 1).

All other hierarchical regression models for inter-sentential and intra-sentential switching in the naturalistic conversation task on verbal and non-verbal cognitive control processes were non-significant (all *p*s > 0.05). This demonstrates that bilinguals’ inter- and intra-sentential switching behaviors were not predictive of other verbal and non-verbal cognitive control processes.

## Discussion

In this study, the relationship between language switching engagement and cognitive control was examined according to the assumptions and predictions of the Adaptive Control Hypothesis (Green and Abutalebi, 2013). The model stands out as a widely referenced bilingual language control model, but it has had mixed support. No study to our knowledge has comprehensively tested the set of assumptions and predictions, and most previous work examined self-reported engagement in language contexts as related to cognitive control. In the current study, both self-report measures and observed behavior measures of language switching were collected, to gain a fuller understanding of language control history and performance as related to cognitive control. This allowed us to examine more directly the predictions of the model.

First, we examined the model’s assumption that there are three types of interactional bilingual contexts in which bilingual individuals may engage. We considered whether their engagement is exclusive to one context, or alternatively that their engagement across context types may be fluid. We then tested predictions about primary engagement in the single, dual, or dense code-switching language contexts and language switching behaviors. Next, the relation of individual differences in language switching experience with both verbal and non-verbal measures of cognitive control was also examined. Lastly, this study also examined the model’s assumptions which associate the production of different types of language switches with verbal and non-verbal cognitive control.

### Individual Variations in Bilingual’s Reported Engagement in the Three Interactional Contexts

Findings from this study showed that self-reported engagement in the single-language context was negatively and weakly correlated with engagement in the dual-language context and with general language switching. However, positive and moderately strong correlations were observed between engagement in the dual-language context and inter-sentential switching, and with the dense code-switching context (intra-sentential switching). These findings could suggest that there is fluidity between bilinguals’ engagement in the three interactional contexts. The distinction of these interactional contexts could be less pronounced in a multilingual environment, where bilingualism is prevalent and multiple languages are widely present and used.

The single-language context was measured through the degree in which bilinguals speak one language in one environment and frequency of language switching. Engagement in the dual-language context was measured through the tendency to speak both languages in the same environment and switch languages between sentences during conversation (inter-sentential switching). In this current study, the more bilinguals report engaging in a linguistic environments where their languages are used and kept separate (i.e., monolingual mode), the less likely they are to engage in linguistic environments where they are exposed to both languages and switch between them frequently (i.e., bilingual modes). However, the smaller correlations (*r* = −0.29, *p* < 0.05) could suggest a lack of clear distinction between primary engagement in a single-language context and dual-language context (i.e., they are not diametrically opposite to one another). These findings suggest that there could be fluidity in engagement between these two interactional contexts, especially within more multilingual populations.

This is further observed between the dual-language context and dense code-switching context. Findings from this study suggest that higher reported engagement in dual-language context is positively associated with the dense code-switching context. Dense code-switching was measured through the inclusion of words from one language into the other (intra-sentential switching) when conversing with others. These findings suggest that bilinguals who report higher engagement in a dual-language context and inter-sentential switching, also report higher intra-sentential switching. This supports current evidence that bilinguals tend to produce both types of language switches naturally especially under voluntary language switching contexts (e.g., Blanco-Elorrieta and Pylkkänen, 2018; Yow et al., 2018). This also highlights the fluidity between the dual-language context and dense code-switching context. In relation to the Adaptive Control Hypothesis, although the distinction of the three interactional contexts are proposed, it also acknowledges that there may be fluidity in bilinguals’ linguistic environments. Current findings lend support to the model’s view of linguistic fluidity and could suggest that the model should present the three interactional contexts on a continuum instead.

In this study, we observed that all bilingual participants reported being regularly exposed to both languages, and to using and switching between them to varying extents (see Tables 3, 5). This could suggest that bilinguals, especially in multilingual societies, may not categorically find themselves in a single type of interactional context. This could highlight that there is fluidity in their linguistic environments, and where there is frequent exposure and use of both their languages. This reflects current views which advocate that bilingualism is a dynamic experience and not a categorical variable (e.g., Luk and Bialystok, 2013). While it is theoretically assumed within the Adaptive Control Hypothesis (Green and Abutalebi, 2013) that the single-language context (one language in one environment, and low frequency of language switching) is the opposite of the dual-language context (both languages in one environment, with high frequency of controlled language switching), a clear distinction of primary engagement in these interactional contexts might not be possible within multilingual populations. Although bilinguals might generally find themselves in linguistic contexts where there is relatively higher separation of their languages and may switch less regularly (single language context), it is still highly likely that they are still exposed to both languages and may use both languages to a certain degree on a regular basis. This is particularly observed with the dual-language context and dense code-switching context. While both groups of bilinguals are proposed to be highly exposed to both languages and switch between them regularly, the model proposes that they differ in the way that they switch between their languages (e.g., intra-sentential switching of dense code-switchers). However, current findings show that these contexts may not be clearly distinguishable.

Within the hypothesis, it is alternatively proposed that bilinguals may not find themselves distinctly in each of these interactional contexts due to the fluidity in bilinguals’ natural communicative environments and linguistic ecologies. Current findings support this alternative view and suggest that the ecological validity of the three interactional contexts might not be clearly applicable in more multilingual populations where language experiences may overlap. This might suggest that bilinguals’ linguistic ecologies cannot be categorically defined or operationalized, and should be presented on a continuum. It can be argued that attempts to categorically classify the types of linguistic contexts, could cloud the actual dynamism and complexities that may occur in bilinguals’ natural linguistic ecology.

### Individual Variations in Bilingual’s Reported Engagement in the Three Interactional Contexts and Observed Language Switching Behaviors

In order to examine the self-reported contextual categories, we compared observed measures of language switching across different language switching task constraints and how these related to one’s reported language context. For example, it would be expected that individuals reporting higher engagement in dense code-switching context, which includes self-rating items of intra-sentential switching, would also show more intra-sentential switching when objectively measured. Findings from this study showed that self-reported engagement in the three interactional contexts was not correlated with any type of observed language switching behaviors (word switching, inter- and intra-sentential switches) (Table 9). How bilinguals engage in their language environments on a day-to-day basis does not seem to be related to how they produce and switch between their languages in their immediate language environment. This challenges the Adaptive Control Hypothesis in its assumption which associate bilinguals’ engagement in different interactional contexts with their language switching behaviors.

To measure and categorize bilinguals’ primary engagement in the three types of interactional contexts, six survey items were selected from the Language Switching Questionnaire (refer to the Section “Classification of Bilinguals in the Three Interactional Contexts” for details on classification of interactional contexts). The single-language context was measured through the extent that bilinguals keep their languages apart and the extent that they use both languages in the same environment (reverse coded) and engage in general switching (reverse coded). Dual-language context was measured through the extent that both languages are spoken in the same environment, and frequency of switching between sentences when conversing with others (i.e., inter-sentential switching). Dense code-switching was measured through the extent that bilinguals include words and phrases from one language (e.g., Chinese or English) into the other when they converse with others. Each participant had three scores, with each score reflecting the extent of engagement in each interactional context.

While the model’s construct of the different interactional contexts may be theoretically helpful, current measures in this study and to date are defined through subjective self-report measures. These measures may not distinguish categorically between qualitatively different life experiences. This is of particular consideration given the observed fluidity of bilinguals’ engagement in different interactional contexts. Accordingly, attempts to categorize a fluid and continuous experience such as language switching, through subjective self-reported measures, might be less applicable and challenging in more multilingual populations due to the overlap of language experiences. This could limit the extent to which differences in language switching behaviors might be observed.

Further, there is evidence that bilinguals’ language switching behaviors are contextually and environmentally dependent. The types of language switches that are produced spontaneously could be dependent on immediate factors such as the communicative contexts (e.g., cues and language demands) they are in and intentions for switching, rather than on regular language usage (e.g., Blanco-Elorrieta and Pylkkänen, 2017). In view of the fluidity of bilinguals’ engagement in different language environments, bilinguals in this study may also be adept at switching between their languages based on varying factors and cues. As such, how bilinguals produce and switch between their languages may be dependent on immediate environmental factors instead. However, these factors are not discussed within the Adaptive Control Hypothesis or this study. Due to the observed dearth of studies that have examined why (intention) and when (context) bilinguals may switch between their languages, the factors that may influence the types of language switches bilinguals produce in their immediate linguistic environment, is unclear.

Current findings could call into question the distinct types of interactional contexts, especially in multilingual populations where there is fluidity in their linguistic environments. It could also demonstrate the methodological difficulty in neatly categorizing bilinguals based on the types of language switching behaviors that they subjectively report to produce on a day-to-day basis. In view that language switching behaviors might be contextually dependent, this could also challenge the model’s assumption that associates bilinguals’ primary engagement in different interactional contexts with specific language switching behaviors. Future research will need to re-examine the classification of these interactional contexts and methodological approaches to measure them, to better reflect the natural language ecology of bilinguals (e.g., fluidity of communicative environments) and its influence on language switching behaviors. Future research should also focus on examining the factors that influence how bilinguals switch between their languages in their immediate linguistic environment.

### Relationship Between Bilinguals’ Degree of Engagement in Interactional Contexts With Verbal and Non-verbal Cognitive Control

In this current study, regression analyses revealed that after accounting for bilingual proficiency, bilinguals’ reported frequency of engagement in a dual-language environment showed a tendency to account for additional variance in engagement and disengagement for verbal control (smaller switch cost in the Stroop task) (Table 13). These findings may lend support to the Adaptive Control Hypothesis that higher engagement in a dual-language context could confer cognitive control efficiency especially in the verbal domain, though the impact was not strong and only marginally significant. This contributes to a growing narrative, in demonstrating the positive association between engaging in linguistic environments in which bilinguals have to frequently use and switch between their languages on a daily basis, with cognitive control efficiency (e.g., Hartanto and Yang, 2016; Henrard and van Daele, 2017).

Engagement in the dual-language context was measured through the tendency to speak both languages in the same environment and switch languages between sentences during conversation (inter-sentential switching). Cognitive verbal efficiency in engagement and disengagement could be conferred due to the high presence and use of both languages in their daily interactions, where bilinguals have to constantly activate and switch (engage and disengage) between their languages. In order to communicate across different linguistic contexts, they have to select the appropriate representation of the target language, inhibit the non-target language, and switch between them (Green and Abutalebi, 2013). This simultaneous activation of linguistic competition between different languages could cause competition and necessitate bilinguals to engage and disengage from one language to another. As such, increased engagements in a dual-language environment could “train up” control processes related to verbal engagement and disengagement, leading to greater cognitive control efficiency. In view of its significance, future research should closely examine bilinguals’ language environments, to identify the environmental conditions that may enhance language and cognitive efficiency.

However, higher reported engagement in the dual-language context did not predict performance on other cognitive control processes. This does not support the Adaptive Control Hypothesis associating higher engagement in a dual-language context with domain-general cognitive control processes. These findings could be attributed once again to the fluidity of bilinguals’ language environments. In multilingual societies such as the one in which this study took place, bilinguals are constantly exposed to environments in which both languages are frequently present. Bilinguals may use their languages interchangeably and regularly as part of their normal communicative exchanges. As observed, all bilingual participants in this study reported being regularly exposed to both languages and to using and switching between them to varying degrees (Table 3). As such, the extent of engagement in a dual-language context on domain-general cognitive control processes might not be distinctively and adaptively observed especially when examined within multilingual populations who engage in dual-language environments and use both their languages regularly.

Perhaps the categories of interactional contexts, as defined and measured here, are too broad, and more fine-scaled measures of “intensity” of engagement is required. Future studies can consider examining a threshold of engagement within bilingual linguistic environments, to determine if a certain intensity is required before cognitive effects might be observed. It would also be informative to examine within bilingual environments to further identify key aspects of bilingual environments and how bilinguals switch and use their languages. Future research can also investigate how variations in these aspects might influence cognitive control over time.

### Relationship Between Bilinguals’ Naturalistic Language Switching With Verbal and Non-verbal Cognitive Control

In examining bilinguals’ word and sentential language switches, when objectively measured, a more nuanced and complex relationship with cognitive control is revealed. Findings from various regression analyses reveal the distinct association between naturalistic language switching behaviors and verbal and non-verbal cognitive control. In this study, higher frequency of inter-sentential switches (controlled language switching) in the naturalistic conversation task (i.e., uncued switching and high ecological validity) predicted efficiency in non-verbal goal maintenance (Global-Local task: Faster RTs for local incongruent trial types in mixed block) (Tables 14 and 15).

In the naturalistic conversation task, language switching is argued to be voluntary and is uncued (Declerck and Philipp, 2015). In this task, bilinguals’ use of their languages is internally driven (i.e., not based on external rules or cues). Bilinguals can choose voluntarily how they want to use and switch between their languages, and language switches may be produced for the ease of communication (e.g., Blanco-Elorrieta and Pylkkänen, 2017, 2018; de Bruin et al., 2018). Findings from this study suggest that in such a linguistic task, the voluntary production of controlled sentential language switches (inter-sentential switching) is related to efficiency in non-verbal goal maintenance and interference control (conflict monitoring). This could lend support to the Adaptive Control Hypothesis and previous theoretical views that propose higher cognitive control to be associated with controlled types of sentential language switches (e.g., Muysken, 2000; Treffers-Daller, 2009; Green and Wei, 2014). Within the literature of sentential language switching types, inter-sentential switching (also known as alternation) is when bilinguals alternate between structurally independent stretches of two languages (Muysken, 2000). Based on the idea that greater language separation equates to greater cognitive control, inter-sentential switching is implied to involve high cognitive control (e.g., interference control) due to the active suppression required in language use. This corroborates with previous evidences, which have found that self-reported frequency of engagement in inter-sentential switching is associated with non-verbal cognitive efficiency (e.g., Hartanto and Yang, 2016). This study extends current knowledge, by associating higher linguistic control (inter-sentential switching) with efficiency in non-verbal goal maintenance and interference control (conflict monitoring) processes.

In this study, cognitive control processes proposed within the Adaptive Control Hypothesis were examined through variations of verbal and non-verbal input. Current findings could suggest that bilinguals’ language control, especially when it is driven internally and voluntarily, implicates distinct domains of cognitive control (e.g., non-verbal) and cognitive control processes (e.g., goal maintenance and conflict monitoring). This shows the cognitive complexity of bilinguals’ naturalistic language switching production and the diversity of the cognitive control network (Miyake and Friedman, 2012). Current findings indicate that there are different types of language switches, where its varieties differ in its effects on cognitive processing. These varieties have corresponding implications for control processes that are assumed to be involved (e.g., Muysken, 2000; Treffers-Daller, 2009; Green and Abutalebi, 2013; Green and Wei, 2014). It highlights that different types of cognitive information are managed and processed depending on how bilinguals engage in language control (internally or externally) and switch between their languages. This advances the current understanding of the distinct interaction between language switching behaviors and cognitive control. Future research should focus on examining the distinction between verbal and non-verbal cognitive control, particularly in relation to language switching behaviors. Future research should also examine the nature of language control engagements (e.g., internally vs. externally driven) and understand its association with cognitive control processes.

## Limitations

The current examination of the Adaptive Control Hypothesis’ assumptions and predictions was intended to be comprehensive. However, findings should be interpreted in light of several limitations. The first is methodological, in terms of the measures of individual differences in bilingual experiences. We included more objective measures of language control alongside more traditional self-report measures. However, the language switching tasks were experimental tasks taking place in a lab. Even more ecologically valid approaches could overcome the present contextual limitations in how bilinguals may naturally use and switch between their languages. This is particularly pertinent in measuring the naturalistic production of language switching behaviors as it might not fully reflect bilinguals’ naturalistic use of their languages on a daily basis.

Another consideration for the generalizability of the current results is regarding the socio-linguistic context and profile of the study’s participants. The study took place in a multilingual society, where mixing of various ethnic languages is widespread. This may differ from other social contexts in which there is greater language separation. Also, the sample was restricted to one bilingual group (English–Mandarin young adult bilinguals). The results of this study may not be fully generalizable to other groups of bilinguals with different language pairings (e.g., languages types that are more similar or different) (see Coderre and van Heuven, 2014). Future research could examine the model in other populations, particularly those where there is a greater degree of language separation between one’s available languages.

The next consideration is that this study did not assess individual variables such as non-verbal intelligence as control variables. As participants in this study were from an educationally homogenous population (i.e., public tertiary educational institution). Other control variables such as age and bilingual proficiency were deemed to be relevant control variables within such a population. However, the potentially contributing effects of individual variations in other aspects of cognition on cognitive control performance cannot be ruled out based on this current study.

## Conclusion

Through the use of multiple language switching measures that include objective, rigorous, and naturalistic tasks, results from the current study showcase the multi-dimensionality of language switching and its complex interaction with cognitive control processes. This study is novel and important in extensively examining the assumptions and predictions of the Adaptive Control Hypothesis collectively (Green and Abutalebi, 2013). Overall, the hypothesis is supported only to a certain extent. Findings suggest that the distinct classification of the three types of interactional contexts might not be as clearly distinguishable especially in more multilingual populations. Instead, there may be fluidity in bilinguals’ communicative contexts, and bilinguals may find themselves engaging in each of these different contexts to varying degrees. The model’s assumption that associates bilinguals’ primary reported engagement in their language environments with language control was not observed this study. This could further highlight the notion of fluidity of bilinguals’ engagement in their language environments and suggest the difficulty in categorizing such a fluid and continuous experience (i.e., language switching) through current self-report measures. Findings could also highlight that there might be other factors that may influence how bilinguals switch between their languages in their immediate linguistic environment. From this study, the distinct relationship between bilingual s naturalistic language switching behaviors and cognitive control processes is observed. This suggests the complex and distinct interaction between bilinguals language control and cognitive control.

A strength of the Adaptive Control Hypothesis model is that it provides explicit predictions about adaptive and distinct cognitive effects associated with bilinguals’ primary engagement in the different interactional contexts. However, not all of these predictions were supported—with only one trend for verbal cognitive control. Support for the hypothesis is noted in the cognitive efficiency associated with observed language switching behaviors (i.e., language control). Current findings associate high language control, as reflected through the naturalistic verbal production of word switching and inter-sentential switches, with both verbal and non-verbal cognitive efficiency. In conclusion, this study is significant in examining the Adaptive Control Hypothesis, and it brings us a step closer in understanding the intricate relationship between language switching engagements and different domains of cognitive control processes.

## Data Availability Statement

The datasets generated for this study are available upon request to the corresponding author.

## Ethics Statement

The studies involving human participants were reviewed and approved by Nanyang Technological University and Temasek Polytechnic. Written informed consent from the participants’ legal guardian/next of kin was not required to participate in this study in accordance with the national legislation and the institutional requirements.

## Author Contributions

Both GL and BO’B were involved in the design and development of this entire study.

## Conflict of Interest

The authors declare that the research was conducted in the absence of any commercial or financial relationships that could be construed as a potential conflict of interest.
